# 1890. Evaluating the effect of respiratory isolation for TB on patient- and public health-important outcomes- a systematic review

**DOI:** 10.1093/ofid/ofad500.1718

**Published:** 2023-11-27

**Authors:** Abarna Pearl, Amanda M Biewer, Naveed Delrooz, Advaith Subramanian, Sarah E Miller, Leonard Mukasa, Laura R Young, Sundari R Mase, Sonal Munsiff, Edward Nardell, Ruvandhi Nathavitharana

**Affiliations:** Beth Israel Deaconess Medical Center, Boston, Massachusetts; Beth Israel Deaconess Medical Center, Boston, Massachusetts; Tulane University, San Carlos, California; Tulane University, San Carlos, California; N/A, Oklahoma City, Oklahoma; Department of Health, Arkansas, Little Rock, Arkansas; Virginia Department of Health, Richmond, Virginia; Stop TB USA, Atlanta, Georgia; Univ. of Rochester, Rochester, NY; Brigham and Women's Hospital, Boston, Massachusetts; Beth Israel Deaconess Medical Center, Boston, Massachusetts

## Abstract

**Background:**

The duration of respiratory isolation for infectious tuberculosis (TB) is based on limited data and expert opinion. Yet the impact of isolation on persons with TB and public health programs is significant. This systematic review synthesized evidence on public health and patient-important outcomes of respiratory isolation for TB to inform revised National TB Controllers Association guidelines.

**Methods:**

We searched PubMed, EMBASE, CINAHL, Web of Science, Cochrane Central and WHO-Global Index Medicus using terms for TB and respiratory isolation (Figure). Eight reviewers screened abstracts, full-texts, and extracted data in pairs. Inclusion criteria were data on effects of respiratory isolation compared to no isolation or masking. Studies were stratified by outcomes: TB infection or TB disease in contacts, mortality, hospitalization duration, patient and health system costs, and impact on mental health or stigma. A convergent mixed methods approach was used to integrate quantitative and qualitative findings and assess limitations.

**Figure:**
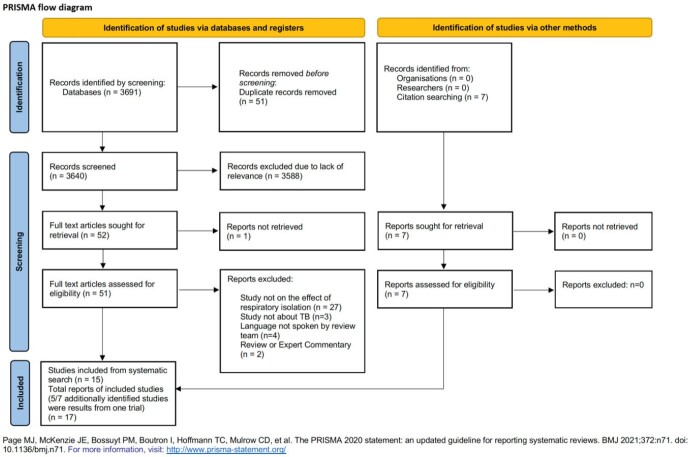

**Results:**

After screening 3640 publications, 17 studies: randomized controlled trial (1), quasi-experimental (1), cohort (1), modelling (3), mixed-methods studies (3), and qualitative (8), were included. The trial (conducted in the 1950s) suggested treatment in isolation in a sanatorium versus at home did not affect TB infection (22% versus 23%) or disease incidence (11% versus 10.5%) in contacts (Table 1). Modelling studies suggest isolation may reduce transmission, including drug-resistant TB, but highlighted isolation is rarely implemented without other interventions, including treatment or masking. Many studies described adverse impacts of isolation on employment, education, food/housing security, and mental health due to transmission fears, stigma and social isolation (Tables 2 & 3). Impacts were compounded in marginalized groups such as indigenous and incarcerated persons.

**Figure:**
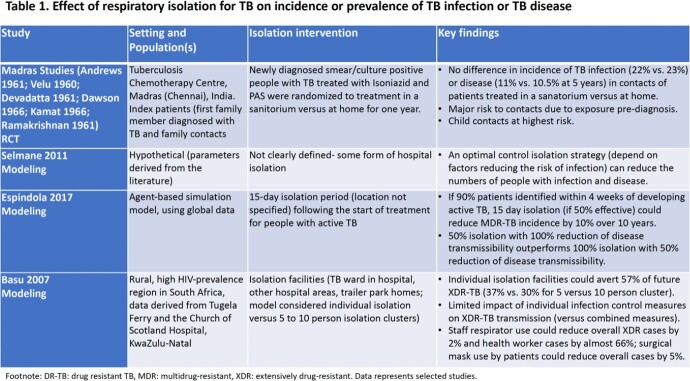

**Figure:**
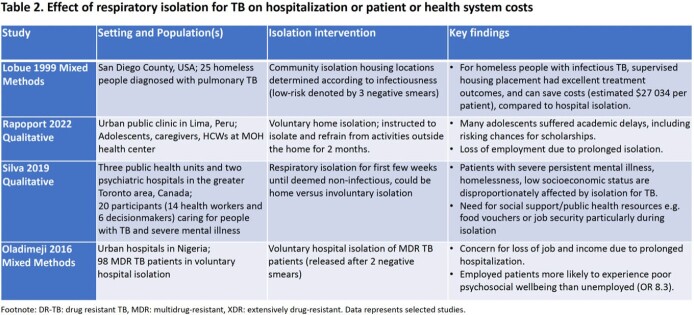

**Figure:**
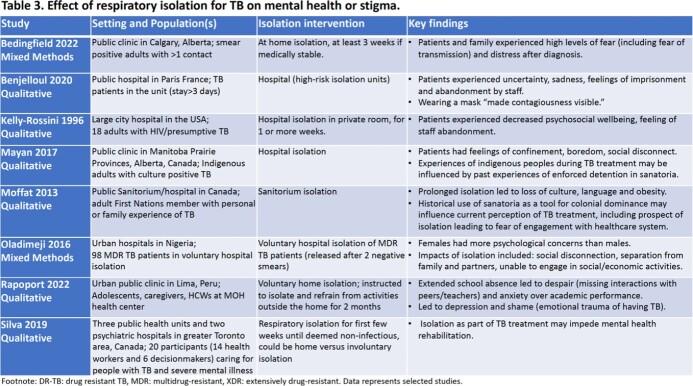

**Conclusion:**

Data to support current isolation practices, particularly once effective treatment is started, to reduce TB transmission in communities are limited. Public health guidance should consider the negative impacts on persons with TB against the potential for transmission reduction to facilitate evidence-based decisions about respiratory isolation.

**Disclosures:**

**All Authors**: No reported disclosures

